# Study on Spatiotemporal Variation in Internal Temperature Field in Quartz Flexible Accelerometer

**DOI:** 10.3390/mi16020241

**Published:** 2025-02-19

**Authors:** Zhigang Zhang, Fangxiang Tang, Ziwei Zhao, Zhao Zhang, Lijun Tang

**Affiliations:** 1 School of Physics and Electronic Science, Changsha University of Science and Technology, Changsha 410114, China; zhangzg@csust.edu.cn (Z.Z.); 24111032126@stu.csust.edu.cn (F.T.); zhangzhao@csust.edu.cn (Z.Z.); 2 Hunan Provincial Key Laboratory of Flexible Electronic Materials Genome Engineering, Changsha 410114, China; 3 Key Laboratory of Electromagnetic Environment Monitoring and Modeling in Hunan Province, Changsha 410114, China; 4 School of Materials and New Energy, Ningxia University, Yinchuan 750021, China; zhaozw@nxu.edu.cn

**Keywords:** quartz flexible accelerometer, heat transfer simulation model, unsteady heat conduction, temperature field

## Abstract

Quartz flexible accelerometers (QFAs) are a type of temperature-sensitive sensor, whereby a change in temperature will cause the key parameters of the accelerometer to drift and cause stability errors. Due to the absence of effective methods for sensing the temperature of internal accelerometer components, existing temperature error correction approaches primarily rely on shell temperature measurements to establish correction models. Consequently, most correction methods achieve higher accuracy during the steady-state heat conduction phase of the accelerometer, whereas the correction error markedly increases during the transient heat conduction phase. To elucidate the temperature discrepancy between the QFA shell and its internal components and to support the development of a temperature error correction method for QFAs based on the internal temperature as a reference, this paper investigated the heat exchange dynamics between the interior and exterior of a QFA. A thermal conduction simulation model of the QFA was established, from which the spatiotemporal distribution patterns of the internal temperature field were derived. The results indicate that the temperature of the QFA shell changes significantly faster than that of the internal meter head in the early stage of the temperature change. The temperature gradient between the shell and the meter head first increases and then decreases, and the rate of temperature change in the upper part of the accelerometer is faster than that in the lower part. Before thermal equilibrium is reached, the temperature distribution inside the accelerometer is uneven in terms of time and space. Inside the accelerometer, the yoke iron, swing plate assembly, servo circuit, and magnetic steel assembly are the main components that exhibit differences in the internal temperature change in the QFA. When developing the temperature error correction method, it was crucial to address and mitigate the impact of temperature variations among these components. The average RMSE between the predicted temperature from the heat transfer model established in this paper and the experimental results was 0.4 °C. This indicates that the model can accurately predict the temperature variation within the QFA, thereby providing robust support for investigating the temperature behavior inside the QFA and offering essential technical foundations for enhancing the accuracy of the temperature error correction method.

## 1. Introduction

Inertial navigation systems exhibit high accuracy, strong stealth capabilities, and resistance to external interference. They hold a critical position in modern navigation technology and serve as an indispensable navigation method for various moving platforms, including aircraft, spacecraft, submarines, and rockets. An inertial navigation system primarily consists of two key inertial components: gyroscopes and accelerometers. The performance and accuracy of these devices have a direct impact on the precision of navigation and guidance. Among these, accelerometers can be categorized into several types based on their distinct working principles, including pendulous integrating gyroscopic accelerometers, QFAs, quartz vibrating beam accelerometers, MEMS accelerometers, and atomic interferometric accelerometers [1]. Currently, QFAs have become the preferred accelerometer type for medium- and high-precision navigation due to its significant advantages of high accuracy, small size, light weight, and low power consumption [2]. With the escalating demands for higher navigation and positioning resolution and reliability in industries such as aerospace, autonomous driving, and low-altitude economy, increasingly stringent requirements have been imposed on the accuracy and stability of inertial devices like QFAs.

A QFA measures acceleration by utilizing an internal sensitive mass. When the mass displaces due to acceleration, it induces torque on the flexible support structure. In response, the torque-sensing mechanism generates an electromagnetic force that restores the mass to its equilibrium position. During this process, the change in current is proportional to the magnitude of the acceleration, thereby enabling the precise measurement of acceleration. This measurement process necessitates the precise coordination of multiple components within the accelerometer, involving changes in various physical quantities such as displacement and electromagnetic force. Temperature variations affect the structural dimensions and performance parameters of the internal components due to material thermal expansion and temperature-dependent magnetic induction intensity. Consequently, these changes cause deviations in the physical quantities from their set values, leading to drift in key parameters of the accelerometer (primarily the bias $K_{0}$ and scale factor $K_{1}$), thereby inducing acceleration measurement errors. The inherent temperature sensitivity of QFAs, which stems from their susceptibility to the thermophysical properties of their internal materials, necessitates correction of temperature-induced acceleration measurement errors to enhance measurement accuracy and stability.

Typically, two primary methods are employed to correct temperature errors in QFAs. The first method involves installing an insulating shell around the accelerometer and maintaining a constant temperature within the insulated cavity through adjustments made by a temperature control system. This method circumvents the need to correct for the drift in key accelerometer parameters, but it demands a higher level of temperature control accuracy to mitigate parameter drift induced by temperature variations. To enhance the temperature control accuracy, several researchers have proposed various schemes, including a temperature control algorithm based on Proximal Support Vector Regression (PSVR) [3], a system identification approach for modeling the temperature control object of an accelerometer [4], and a temperature control system utilizing an incremental PID control algorithm [5]. This temperature control method primarily relies on initial theoretical analysis and experimental validation to elucidate the heat generation during accelerometer operation and the variation patterns of heat exchange between the insulating shell and the external environment. Based on these findings, the required heat output for the temperature control system is calculated to maintain thermal equilibrium within the cavity at all times. Typically, temperature fluctuations can be maintained within ±0.5 °C [5]. However, this level of temperature control accuracy is insufficient for high-precision measurement scenarios that are highly sensitive to temperature variations, such as gravity measurements using QFAs. Consequently, researchers have developed a multi-level temperature control system to further minimize temperature fluctuations within the cavity [6]. By implementing stepwise precise temperature control, the fluctuation range can be reduced to within ±0.1 °C [7], significantly enhancing the measurement accuracy of the accelerometer. The installation of insulation shells can significantly enhance the temperature stability of QFAs. However, this method also leads to an increase in the weight and volume of the acceleration measurement system. Moreover, it is challenging to implement in high-temperature environments such as those encountered during drilling operations, thereby limiting its applicability in certain scenarios.

**Figure 1:**
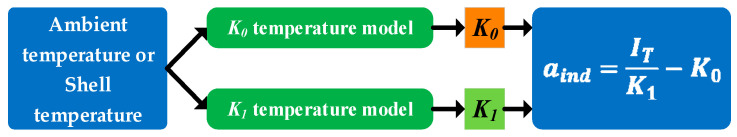
Procedure of the conventional temperature error correction method for QFAs.

Another method to correct the temperature error of QFAs involves establishing a temperature model for the accelerometer’s bias $K_{0}$ and scale factor $K_{1}$. This approach entails obtaining the correction values of $K_{0}$ and $K_{1}$ at various temperatures and subsequently calculating the acceleration indication value $a_{ind}$ after temperature error correction based on the torque generator’s output current $I_{T}$ [8]. The fundamental calculation process is illustrated in Figure 1.

This method does not necessitate additional hardware devices. Instead, it primarily depends on the mathematical correlation between parameters and temperature for error correction, thereby imposing relatively stringent requirements on the precision of the temperature model. The early development of $K_{0}$ and $K_{1}$ temperature models primarily utilized linear fitting methods [9,10]. Over time, these models evolved to employ least-squares fitting for the establishment of nonlinear temperature models based on second-order or higher-order polynomials [11,12,13]. This advancement significantly enhanced the error correction accuracy, resulting in a measurement precision that was an order of magnitude greater than pre-correction levels [14]. This correction method has also been extensively applied to temperature error correction in various types of accelerometers, including MEMS [15]. Subsequently, Yu et al. introduced higher-order terms of the temperature gradient into the high-order temperature model and developed a combined polynomial compensation model based on both the temperature and temperature gradient [16]. This approach significantly enhanced the drift correction accuracy of the parameter $K_{0}$ Liu et al. proposed a dynamic temperature testing method, which revealed the hysteresis in parameter drift of the QFA during both heating and cooling processes. These findings further confirmed the strong correlation between parameter drift and the temperature gradient [17]. The temperature model of QFA parameters based on high-order polynomials was subsequently enhanced by incorporating the temperature gradient term [18,19,20], thereby facilitating the evolution of the temperature model from a single spatial dimension to encompass both spatial and temporal dimensions. In addition to conventional polynomial temperature models, scholars have also proposed advanced temperature models utilizing machine learning algorithms such as particle swarm optimization [21], an integrated algorithm combining improved SCM and ANFIS [22,23], transformation analysis methods like wavelet transform [24,25,26], and long short-term memory networks for temperature error correction [27]. This method has been extended and applied to the temperature error correction of MEMS accelerometers [28]. The newly developed algorithms have demonstrated significant effectiveness in correcting temperature errors, serving as exemplary models for this field. Additionally, some scholars have successfully reduced the temperature sensitivity of accelerometers by optimizing their structure [29,30] and enhancing the manufacturing process [31,32], thereby continuously improving the accuracy of temperature error correction.

**Figure 2:**
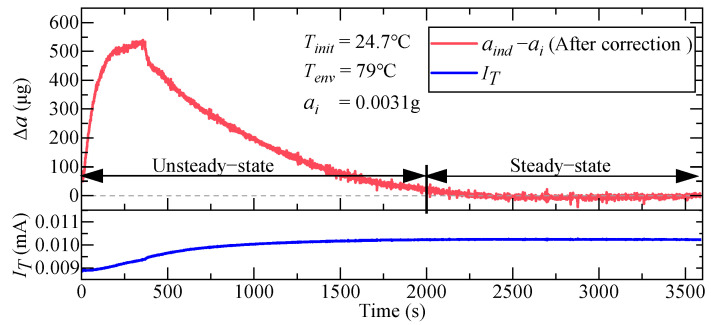
Experimental results of the polynomial temperature correction model.

The aforementioned research significantly improved the correction of temperature-induced errors in QFAs, thereby reducing the impact of temperature on measurement accuracy. Notably, the method of establishing a temperature model for error correction does not require additional hardware, making it easy to deploy and implement. Consequently, this approach has been widely adopted for temperature error correction in accelerometers. However, all these methods rely on steady-state temperature error correction using the external temperature of the accelerometer as a reference. In this scenario, the accelerometer can be approximated as an isothermal object. This approach may compromise the effectiveness of temperature error correction in the temperature model during the non-steady-state heat conduction phase [33]. Taking the widely used polynomial temperature model as an example [34], we conducted experiments to evaluate the error correction effect on the indicated value of a QFA during a rapid temperature increase from 24.7 °C to 79 °C, as shown in Figure 2.

The test results indicate that existing temperature error correction methods exhibit superior accuracy during the steady-state heat conduction phase, whereas the correction error markedly increases during the unsteady-state heat conduction phase. The primary cause of the temperature drift in QFA parameters stems from variations in the internal components rather than the external casing. When a temperature gradient exists between the interior and exterior of a QFA, using the casing temperature as a reference fails to accurately represent the true thermal conditions of the internal components, thereby increasing correction errors [35]. However, due to the absence of precise methods for measuring or predicting the temperature of the internal components of QFAs, current temperature models and algorithms do not account for the spatial distribution of internal temperatures, nor do they incorporate the impact of the temperature gradient among the internal components on the accelerometer parameters [36]. This limitation greatly weakens the measurement stability and reliability of QFAs in complex variable-temperature environments. Therefore, this paper investigates the unsteady heat conduction process of a QFA by analyzing its internal and external heat transfer relationship. This study explores the temporal and spatial distribution patterns of the internal temperature field, establishes an accurate heat transfer simulation model for the QFA, and provides robust technical support for predicting the temperature of internal components and enhancing the temperature error correction accuracy of the QFA in variable-temperature environments.

## 2. Operating Mechanism of QFAs

### 2.1. Architecture and Operational Mechanism of QFAs

**Figure 3:**
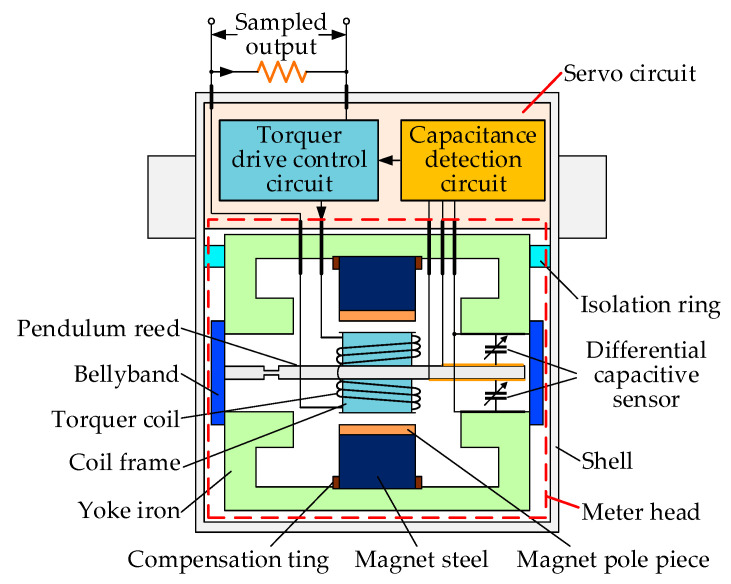
Structure of QFA.

A QFA primarily operates based on Newton’s laws of mechanics. It indirectly measures the acceleration of a moving object by quantifying the inertial force acting on a proof mass. Consequently, this type of accelerometer is also referred to as a specific force sensor. Specific force denotes the net force per unit mass resulting from both gravitational and inertial forces. To measure the specific force acting on the proof mass, a QFA typically employs an electromagnetic force to counterbalance the specific force. Therefore, the magnitude of the electromagnetic force serves as an indicator of the specific force. The interior of a QFA primarily consists of a meter head and a servo circuit, as illustrated in Figure 3. Among them, the meter head is the core component of the QFA, primarily consisting of two major parts: the torquer, which includes the yoke iron, bellyband, magnetic steel, compensation ring, and magnetic pole pieces; and the quartz pendulum assembly, which comprises the tongue-shaped pendulum made of quartz material, the torquer coil, and the coil frame. The torque device generates the electromagnetic force within the accelerometer. The quartz pendulum assembly functions as the proof mass to sense inertial forces, while the servo circuit measures the displacement of the pendulum assembly and regulates the magnitude of the electromagnetic force.

**Figure 4:**
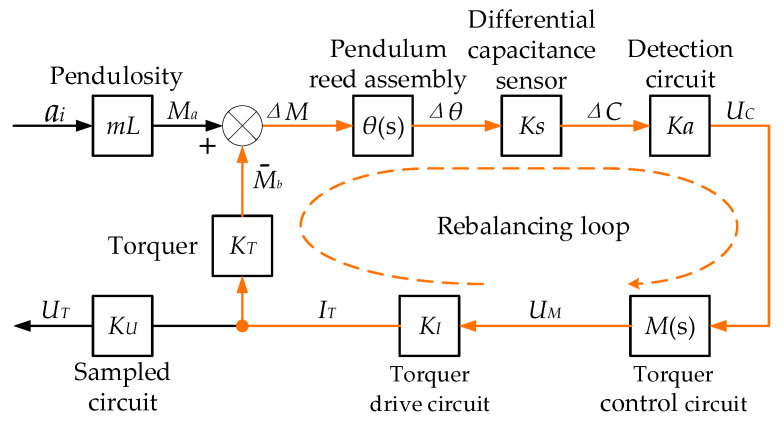
Closed-loop system structure of QFA.

When the QFA is operational, the pendulum assembly functions as the mass element to detect the input acceleration. When the acceleration $a_{i}$ is applied along the input axis of the QFA, the mass unit deviates from its equilibrium position due to the inertial moment $M_{a}$. This deviation causes the mass unit to undergo rotational motion around the flexible pivot, resulting in a deflection angle Δθ. Consequently, this deflection leads to a change in the inter-electrode capacitance of the differential capacitance sensor, generating a differential capacitance ΔC. In the servo circuit, the differential capacitance detection circuit converts the differential capacitance ΔC into a voltage $U_{C}$. This voltage is subsequently transformed into a driving current $I_{T}$ via the torquer drive control circuit, which is then supplied to the torquer coil situated in a constant magnetic field. The electrified torque coil experiences electromagnetic force within the air gap magnetic field of the torque device, generating an electromagnetic feedback torque $M_{b}$ that is equal in magnitude but opposite in direction to the inertial torque $M_{a}$, thereby establishing a force balance. This force balance compels the pendulum sheet assembly to return to its equilibrium position, facilitating closed-loop servo operation. At present, the quartz pendulum assembly, differential capacitance sensor, torquer, and torquer drive control circuit within the structure of the QFA form the rebalancing loop of the closed-loop system. The configuration is illustrated in Figure 4.

In Figure 4, mL is the pendulum property of the pendulum slice assembly, with the unit of kg·m; $\theta \left(s\right)$ is the motion equation of the pendulum slice assembly:

(1) $$\theta \left(s\right)=\frac{1}{JS^{2}+CS+K}$$

where J is the rotational inertia of the pendulum plate assembly around the output shaft, C is the damping coefficient of the pendulum plate assembly, and K is the stiffness of the pendulum plate assembly. $K_{s}$ is the gain coefficient of the differential capacitance sensor, with the unit of pF/rad; $K_{a}$ is the gain coefficient of the differential capacitance detection circuit, with the unit of V/pF; $M\left(s\right)$ is the transfer function of the torque controller system; $K_{I}$ is the gain coefficient of the torque driver circuit, with the unit of mA/V; $K_{T}$ is the scale factor of the torque, with the unit of N·m/mA; and $K_{U}$ is the gain coefficient of the driving current sampling circuit, with the unit of V/mA.

The relationship between the output current of the torque generator in the QFA and the input acceleration can be described as follows:

(2) $$I_{T}=\frac{mL\theta \left(s\right)K_{s}K_{a}M\left(s\right)K_{I}K_{U}}{1+\theta \left(s\right)K_{s}K_{a}M\left(s\right)K_{I}K_{T}}a_{i}$$

In the closed-loop system of the QFA, the torque driver current $I_{T}$ is directly proportional to the input acceleration $a_{i}$. By measuring the output voltage $U_{T}$ from the sampling circuit, the magnitude of the torque driver current $I_{T}$ can be determined, which, in turn, allows for the calculation of the magnitude of the input acceleration $a_{i}$. The direction of $a_{i}$ is determined by the direction of the driving current $I_{T}$.

### 2.2. Critical Parameters of QFAs

**Figure 5:**
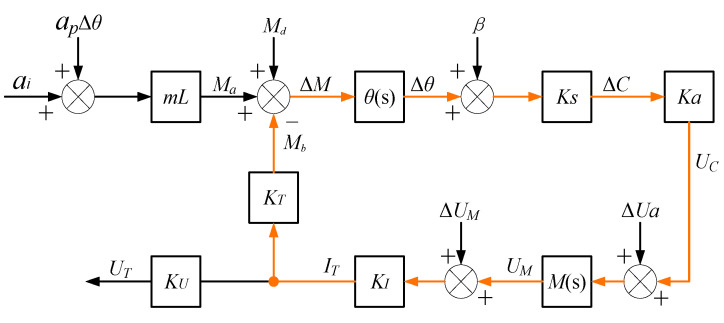
Structure of the closed-loop system for the QFA incorporating the error term.

When processing and assembling a QFA, several errors can occur, including the flatness error of the tongue-shaped quartz pendulum sheet, the uniformity error in the adhesive used for bonding the torque coil, and errors related to the balance and tightness during component assembly. During operation, additional errors, such as zero drift in the servo circuit, may also arise. These unavoidable errors will be introduced into the closed-loop system of the accelerometer, resulting in alterations to its parameters and ultimately leading to acceleration measurement errors. The structure of the closed-loop system of the QFA, including the error terms, is illustrated in Figure 5.

In Figure 2, $a_{p}\Delta \theta$ is the cross-coupling error; $M_{d}$ is the disturbing torque acting on the pendulum assembly around the output axis; β is the elastic recovery angle; $\Delta U_{a}$ is the input disturbing voltage of the differential capacitance detection link; and $\Delta U_{M}$ is the zero output drift disturbing voltage of the torque controller control circuit. The output expression of the torque driver current $I_{T}$ of the QFA, including the error term, is:

(3) $$I_{T}=\frac{mL\theta \left(s\right)K_{s}K_{a}M\left(s\right)K_{I}}{1+\theta \left(s\right)K_{s}K_{a}M\left(s\right)K_{I}K_{T}}\times \left[\left(a_{i}+a_{p}\Delta \theta \right)+\frac{1}{mL}\left(M_{d}+\frac{\beta }{\theta \left(s\right)}+\frac{\Delta U_{a}}{\theta \left(s\right)K_{s}K_{a}}+\frac{\Delta U_{M}}{\theta \left(s\right)K_{s}K_{a}M\left(s\right)}\right)\right]$$

When the system is in a static state, $\theta \left(s\right)=1/K$ and $M\left(s\right)=K_{M}$, where $K_{M}$ represents the static gain of the torque control system. The static formulation of Equation (3) is as follows:

(4) $$I_{T}=K_{1}\left[a_{i}+a_{p}\Delta \theta +\frac{1}{mL}\left(M_{d}+K\beta +\frac{K\Delta U_{a}}{K_{s}K_{a}}+\frac{K\Delta U_{M}}{K_{s}K_{a}K_{M}}\right)\right]$$

where $K_{1}$ is:

(5) $$K_{1}=\frac{mLK_{s}K_{a}K_{M}K_{I}}{K+K_{s}K_{a}K_{M}K_{I}K_{T}}$$

$K_{1}$ is referred to as the scale factor of the QFA, also known as the sensitivity of the accelerometer. Expressed in mA/g, this parameter represents the proportional relationship between the input acceleration and the output current and is a critical characteristic of the QFA.

By dividing Equation (4) by $K_{1}$, the indicated value $a_{ind}$ of the QFA can be obtained as follows:

(6) $$a_{ind}=\frac{I_{T}}{K_{1}}=a_{i}+a_{p}\Delta \theta +\frac{1}{mL}\left(M_{d}+K\beta +\frac{K\Delta U_{a}}{K_{s}K_{a}}+\frac{K\Delta U_{M}}{K_{s}K_{a}K_{M}}\right)$$

The cross-coupling error $a_{p}\Delta \theta$ of a high-performance QFA is typically exceedingly small and can be considered negligible. In the absence of external input acceleration, Equation (6) simplifies to:

(7) $$a_{ind}=K_{0}=\frac{1}{mL}\left(M_{d}+K\beta +\frac{K\Delta U_{a}}{K_{s}K_{a}}+\frac{K\Delta U_{M}}{K_{s}K_{a}K_{M}}\right)$$

$K_{0}$, referred to as the bias value of the QFA (also known as the zero bias or zero output), is measured in units of g. It represents the output of the accelerometer converted into equivalent input acceleration when no external acceleration is present. The bias value $K_{0}$ signifies the inherent output of the accelerometer in the absence of external acceleration and is a critical parameter for characterizing its performance.

Substituting Equation (7) into Equation (4), we obtain the fact that, after incorporating the error term, there exists an approximate linear relationship between the input acceleration $a_{i}$ of the accelerometer closed-loop system and the output driving current $I_{T}$, which is given by:

(8) $$I_{T}=K_{1}\left(a_{i}+K_{0}\right)$$

The aforementioned equation represents the linear model of the QFA. In practical applications, temperature variations affect the accelerometer, leading to temperature-induced drift in the values of $K_{0}$ and $K_{1}$. This drift ultimately results in changes to the output drive current $I_{T}$, thereby causing measurement errors.

## 3. Development of a Thermal Transfer Simulation Model for the QFA

To elucidate the distribution and variation patterns of the internal temperature field within the QFA, this study develops a heat transfer simulation model for a GJN096-D-type QFA, incorporating its assembly structure and material properties.

### 3.1. Analysis of Heat Transfer Mechanisms in the QFA

According to the fundamental principles of heat transfer, there are three primary modes of heat transfer: conduction, convection, and radiation. The primary modes of heat transfer between the QFA and its external environment are conduction through the interface with the fixed base and convective heat exchange between the surrounding air and the shell. The heat transfer mechanisms within the accelerometer encompass three primary modes: conduction, convection, and radiation. Conduction predominantly takes place between components that are in direct physical contact. Thermal convection primarily involves convective heat transfer between the air within the gap among the internal components of the meter head and the surfaces of these components. Thermal radiation primarily originates from the faint thermal emissions produced by the electromagnetic radiation of the torquer coil and the servo circuit. The air gap within the meter head is minimal, resulting in a relatively small temperature differential among internal components. Consequently, heat conduction is the predominant mode of heat transfer within the QFA, while internal heat convection and radiation can be considered negligible. According to the law of conservation of energy, during any time interval, the relationship of heat flow within the accelerometer can be described as follows:

(9) $$Q_{in}+Q_{inter}=Q_{out}+\Delta U$$

where $Q_{in}$ is the heat influx into the QFA. $Q_{inter}$ is the heat generated by the internal heat source within the QFA. $Q_{out}$ signifies the heat efflux from the QFA. ΔU represents the increment in the internal energy of the accelerometer, which can be expressed as:

(10) $$\Delta U=cm\frac{\partial T}{\partial t}=\rho cV\frac{\partial T}{\partial t}$$

where c is the specific heat capacity of the accelerometer material, measured in J/(kg·K). m denotes the mass of the accelerometer; V signifies the volume of the accelerometer; and ∂T/∂t indicates the rate of change in temperature with respect to time.

The internal heat source of the QFA primarily originates from the thermal energy produced by the torquer coil during its operation. The intensity of heat generation from the coil can be quantified as:

(11) $$\dot{\Phi }=\frac{P}{V}=\frac{I_{T}^{2}R_{T}}{V}$$

where $\dot{\Phi }$ is the heat generation intensity of the coil, measured in W/m^3^; P is the heating power of the torque coil; V signifies the volume of the torque coil; $I_{T}$ indicates the driving current of the torque coil; and $R_{T}$ stands for the resistance of the torque coil.

By combining Equations (9)–(11), the temperature field distribution $T_{f}\left(x,y,z,t\right)$ within the QFA during the unsteady heat conduction process can be derived as follows:

(12) $$\frac{\partial T_{f}}{\partial t}=\frac{\partial }{\partial x}\left(\alpha_{x}\frac{\partial T_{f}}{\partial x}\right)+\frac{\partial }{\partial y}\left(\alpha_{y}\frac{\partial T_{f}}{\partial y}\right)+\frac{\partial }{\partial z}\left(\alpha_{z}\frac{\partial T_{f}}{\partial z}\right)+\frac{\dot{\Phi }}{\rho c}$$

where $\alpha_{x}$, $\alpha_{y}$, and $\alpha_{z}$ are the thermal diffusion coefficients in the x, y, and z directions of the accelerometer, respectively, with units of m/s^2^. The thermal diffusion coefficient α can be expressed as:

(13) $$\alpha =\frac{\lambda }{\rho c}$$

In this paper, the heat transfer between the outer surface of the QFA and the surrounding air is modeled as a convective boundary condition, which corresponds to the third type of boundary condition. When the air surrounding the QFA is subjected to forced flow, the heat transfer between the QFA and the surrounding air is characterized as external forced convective heat transfer. The boundary condition is determined using the empirical correlation for fluid flow across a single cylinder:

(14) $$Nu=CRe^{n}Pr^{1/3}$$

where Nu is the Nusselt number, Re is the Reynolds number, and Pr stands for the Prandtl number. The constant C and the exponent n are determined based on the value of the Reynolds number [37]. The Reynolds number (Re) is defined by the following expression:

(15) $$Re=\frac{\rho ul}{\nu }$$

where ρ is the density of the fluid, u denotes the flow velocity of the fluid, ν signifies the kinematic viscosity of the fluid, and l is the characteristic length, which is measured in meters (m). The kinematic viscosity of the fluid and the Prandtl number are determined by the thermophysical properties of dry air, with the qualitative temperature $T_{m}$ being the arithmetic mean of the ambient temperature $T_{\infty }$ and the wall temperature $T_{w}$.

The natural convective heat transfer between the QFA and its surrounding environment can be quantified using the correlation formula for natural convection:

(16) $$Nu=C{\left(Gr\cdot Pr\right)}^{n}$$

where Nu is the Nusselt number, Gr represents the Grashof number, and the product of Gr and Pr is defined as the Rayleigh (Ra) number. The calculation formula for the Gr is as follows:

(17) $$Gr=\frac{g\alpha_{V}\Delta Tl^{3}}{\nu^{2}}$$

where g is gravitational acceleration; $\alpha_{V}$ is the volume expansion coefficient; ΔT is the temperature gradient between the wall temperature $T_{w}$ and the ambient temperature $T_{\infty }$; ν signifies the kinematic viscosity of the gas; and l is the characteristic length.

**Figure 6:**
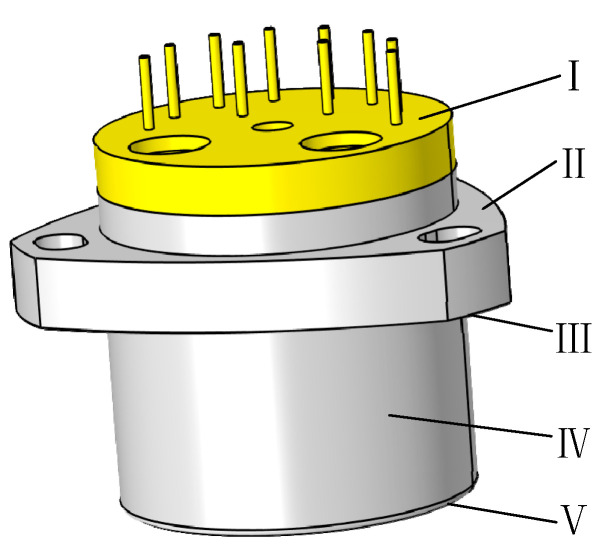
The numerical indication on the external surface of the QFA.

The geometry of the QFA was simplified and abstracted into the shape illustrated in Figure 6. The surfaces of the accelerometer were labeled with Roman numerals I–V, and corresponding experimental correlation equations for each surface were established.

For surfaces No. I and No. II of the QFA, the experimentally derived correlation equation with the horizontal heated surface facing upward is:

(18) $$\left\{\begin{array}{l} Nu=0.54Ra^{1/4},\;10^{4}\leq GrPr\leq 10^{7}\\ Nu=0.15Ra^{1/4},\;10^{7}\leq GrPr\leq 10^{11} \end{array}\right.$$

For surfaces No. III and No. V of the QFA, the experimentally derived correlation formula for the horizontal hot surface facing downward is:

(19) $$Nu=0.27Ra^{1/4},\;10^{5}\leq GrPr\leq 10^{11}$$

Surfaces No. I, II, III, and V of the QFA are all plate heat exchanger, and the characteristic length can be expressed as:

(20) $$l=\frac{A_{p}}{P}$$

where $A_{p}$ is the area of the surface, while P is the perimeter length of the surface.

The surface of accelerometer IV is approximately cylindrical, with a diameter d of 25.40 mm and a height H of 25.40 mm. The height H serves as the characteristic length of the cylinder. Given these dimensions, the surface can be classified as a slender vertical cylinder. The experimental correlation formula from reference [38] is adopted as follows:

(21) $$\left\{\begin{array}{l} \frac{Nu}{Ra^{1/4}}=0.59+0.52\;{\left(Ra^{1/4}\cdot \frac{d}{H}\right)}^{-1},\;0.1\leq Ra^{1/4}\cdot \frac{d}{H}\leq 32\\ \frac{Nu}{Ra^{1/4}}=0.95\;{\left(Ra^{1/4}\cdot \frac{d}{H}\right)}^{-0.79}\;,\;0.0006\leq Ra^{\frac{1}{4}}\cdot \frac{d}{H}\leq 0.1 \end{array}\right.$$

Nu is determined using the correlation formula derived from convective heat transfer experiments. Subsequently, the surface heat transfer coefficient h can be obtained as follows:

(22) $$h=\frac{\lambda Nu}{l}$$

where λ is the thermal conductivity coefficient of the air surrounding the QFA, and l is the characteristic length.

To simulate the rapid changes in ambient temperature around the QFA, the device was rapidly moved in and out of the temperature chamber during the experiment. This procedure ensured that the accelerometer experienced abrupt temperature variations. The rapid movement of the QFA and the frequent opening and closing of the temperature chamber led to both forced and natural convective heat transfer occurring simultaneously between the QFA and its surrounding environment, a phenomenon known as mixed convection. The Nusselt number in the case of mixed heat transfer ($Nu_{M}$) can be expressed as [37]:

(23) $$Nu_{M}=\sqrt[{3}]{Nu_{F}^{3}\pm Nu_{N}^{3}}$$

where $Nu_{F}$ and $Nu_{N}$ represent the Nusselt numbers for forced convective heat transfer and natural convective heat transfer conditions, respectively. When the flow directions are aligned, the term in the formula assumes a positive sign; conversely, when the directions are opposed, it assumes a negative sign. When conducting the rapid temperature change experiment of the QFA, upon completion of the QFA’s movement, the airflow velocity surrounding it gradually decreased, leading to a reduction in $Nu_{F}$. Furthermore, the wall temperature $T_{w}$ of the QFA gradually approached the ambient temperature $T_{\infty }$, reducing the temperature gradient between them, which, in turn, led to a gradual decrease in $Nu_{N}$. Therefore, the Nusselt number ($Nu_{M}$) associated with the mixed heat transfer gradually decreased over time. At this point, the surface heat transfer coefficient h of the QFA, which is defined as a function of time, can be expressed as:

(24) $$h\left(t\right)=\left(h_{0}-h_{\infty }\right)k_{h}e^{-\frac{t}{\tau }}+h_{\infty }$$

where $h_{0}$ is the surface heat transfer coefficient at the initial time (t = 0). $h_{\infty }$ is the surface heat transfer coefficient at thermal equilibrium. $k_{h}$ is the correction factor. τ signifies the time constant associated with the attenuation of the surface heat transfer coefficient, measured in seconds (s).

### 3.2. Development of a Heat Transfer Simulation Model for the QFA

The differential equation (Equation (12)) that describes the distribution of the internal temperature field of the accelerometer typically does not yield an exact analytical solution. Instead, an approximate solution can be obtained through numerical simulation methods. In this paper, a heat transfer simulation model of the QFA was developed using the thermophysical properties of its internal materials. The temperature field distribution and variation within the QFA were analyzed and calculated through finite-element simulations conducted with COMSOL Multiphysics version 6.1. The primary structural parameters of the QFA heat transfer simulation model are detailed in Table 1. The thermal physical property parameters of the materials comprising each component of the QFA are presented in Table 2.

The volume of the torquer coil of the QFA is V = 58.67 mm^3^, and the resistance of the torquer coil is $R_{T}$ = 424 Ω. During the experiment, the maximum acceleration was ±1 g, corresponding to a maximum driving current $I_{T}$ = 1.08 mA and a maximum power consumption P = 4.95 × 10^−7^ W for the torquer. By substituting these values into Equation (11), the heat generation rate of the torquer coil $\dot{\Phi }$ was calculated to be 8429 W/m^3^.

**Figure 7:**
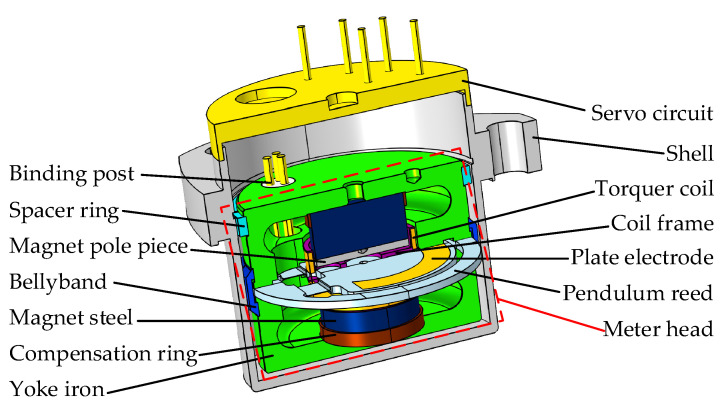
Heat transfer simulation model of QFA.

Based on the aforementioned parameters, a heat transfer simulation model for the QFA was developed, as illustrated in Figure 7.

## 4. Simulation Analysis of Transient Heat Transfer in the QFA

To investigate the temporal variation in the internal temperature field within the QFA, a heat transfer simulation model was utilized. This model calculated the heat conduction process under both increasing and decreasing external environmental temperatures. The simulation calculation accuracy was set to 1%, with a simulation duration of 3600 s. If the surface heat transfer coefficients of each surface of the QFA exhibited minimal variation, they could be reasonably assumed to have a uniform value. The parameters associated with surface heat transfer were set as follows: $h_{0}$ = 33.37 W/(m^2^·K), $h_{\infty }$ = 10.43 W/(m^2^·K), $k_{h}$ = 1, and τ = 130 s.

### 4.1. Analysis of Simulation Results for the Transient Temperature Field of the QFA

**Figure 8:**
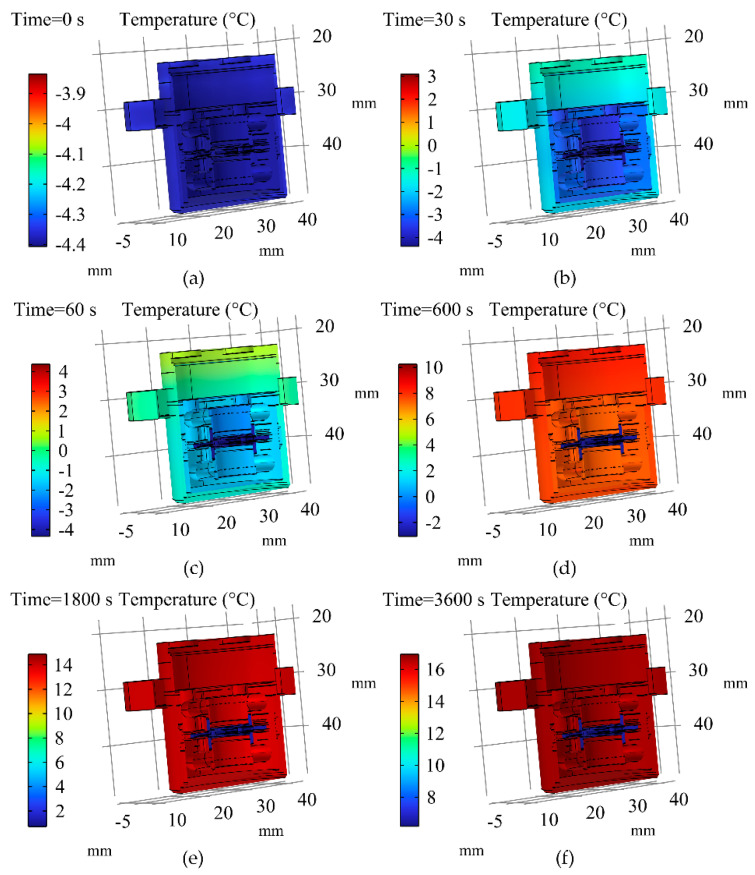
Simulation results of the transient temperature field within the QFA as the ambient temperature increased from −4.3 °C to 17.3 °C. (**a**) Simulation results at 0 s; (**b**) Simulation results at 30 s; (**c**) Simulation results at 60 s; (**d**) Simulation results at 600 s; (**e**) Simulation results at 1800 s; (**f**) Simulation results at 3600 s.

The simulation results of the transient temperature field within the QFA when the ambient temperature increased from −4.3 °C to 17.3 °C are presented in Figure 8. Initially, the temperature field within the accelerometer was uniformly distributed, with all components at the same temperature. As time progressed, the overall temperature of the QFA gradually increased. During the early stages of heating, the outer shell’s temperature rose significantly higher than that of the inner meter head, resulting in a notable temperature gradient between them. Over time, this temperature gradient diminished. In the later stages of heat transfer, apart from the pendulum assembly, the temperature change trends of other internal components aligned with those observed on the QFA’s surface.

**Figure 9:**
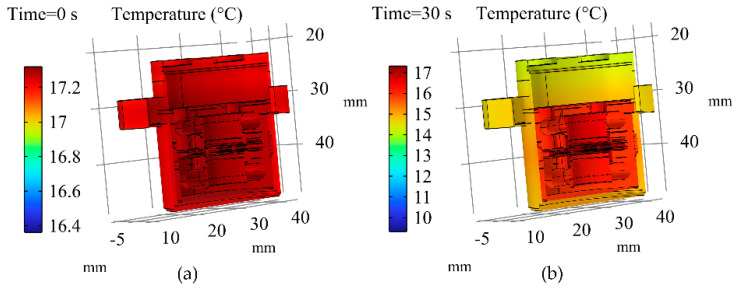

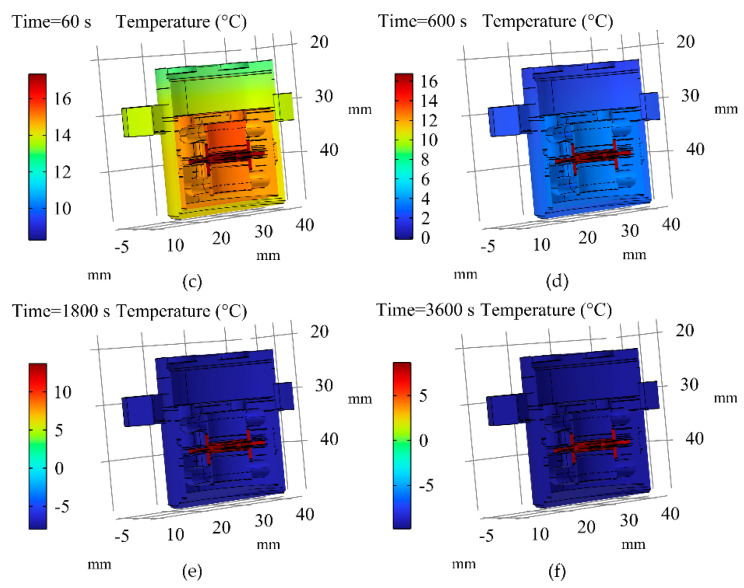
Simulation results of the transient temperature field within the QFA as the ambient temperature decreased from 17.3 °C to −9.7 °C. (**a**) Simulation results at 0 s; (**b**) Simulation results at 30 s; (**c**) Simulation results at 60 s; (**d**) Simulation results at 600 s; (**e**) Simulation results at 1800 s; (**f**) Simulation results at 3600 s.

The simulation results of the transient temperature field within the accelerometer when the ambient temperature decreased from 17.3 °C to −9.7 °C are illustrated in Figure 9. The trend of the internal temperature variation in the accelerometer remained consistent with that observed during an increase in the ambient temperature. In the initial cooling phase, the temperature variation in the outer shell was markedly more pronounced compared to that of the gauge head and diminished over time. As the system approached a steady state, the temperature change trends of the internal components of the QFA became consistent with those of the QFA’s surface.

During the temperature change in the accelerometer, the thermal response of the pendulum assembly was relatively sluggish. This was primarily attributed to the fact that heat conduction occurred exclusively through the flexible beam and support ring, with no direct thermal contact between the pendulum assembly and other components such as the yoke iron and magnetic steel. Additionally, the small dimensions of the flexible beam and the low thermal conductivity of the quartz material further limited the heat transfer. Consequently, the heat flow between the yoke iron, magnetic steel, and the pendulum assembly was minimal, leading to a slower temperature change in the pendulum assembly. Furthermore, the temperature of the upper part of the accelerometer increased more rapidly compared to that of the lower part. This phenomenon primarily occurred because heat in the upper section was mainly transferred from the housing to the servo circuit and subsequently to the air gap. The heat conduction between the servo circuit and the air gap was relatively inefficient, leading to rapid internal energy accumulation within both the servo circuit and the housing, which resulted in a faster temperature rise. In contrast, heat in the lower part was predominantly conducted from the housing into the meter head with higher efficiency. Consequently, during the initial stage, more heat was dissipated from the lower portion of the housing, resulting in a slower internal energy increase and a gradual temperature rise.

### 4.2. Analysis of Temperature Variations Within the Internal Components of the QFA

Using the volume-averaging and surface-averaging functions provided by the simulation software, we obtained the average temperature of the internal components of the QFA and the average temperature of the QFA shell as the environmental temperature varied. Subsequently, the temperature gradient between these two regions was calculated using the following formula:

(25) $$\Delta T_{x}=T_{shell}-T_{x}$$

where x is the identifier for the internal component of the accelerometer; $T_{shell}$ is the average temperature of the shell; $T_{x}$ is the temperature of the internal component; and $\Delta T_{x}$ indicates the temperature gradient between the internal component and the shell. The identifiers for each internal component of the QFA are defined in Table 3.

**Figure 10:**
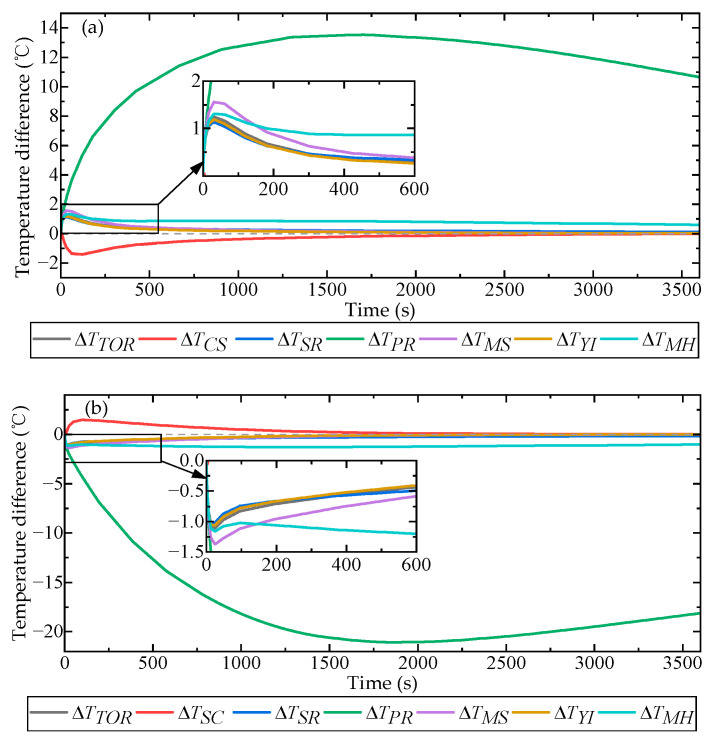
Temperature differential between internal components and shell in response to ambient temperature variations. (**a**) Results observed when ambient temperature rose from −4.3 °C to 17.3 °C; (**b**) results observed when ambient temperature dropped from 17.3 °C to −9.7 °C.

Figure 10 illustrates a curve representing the temperature gradient between the internal components of the accelerometer and the average temperature of the accelerometer housing as the ambient temperature fluctuated over time.

As shown in Figure 10, the changes in $\Delta T_{TOR}$ and $\Delta T_{YI}$ were largely consistent. This was primarily due to the yoke iron being the largest component of the torque device by volume, with a specific heat capacity similar to that of other components such as the magnetic steel. Consequently, the temperature variations between these two components remained largely synchronized. The variations in $\Delta T_{SR}$ and $\Delta T_{YI}$ were essentially identical. This was primarily attributed to the relatively thin thickness of the support ring, which is positioned between the upper and lower yoke irons, resulting in a large contact area with these components. Consequently, the high thermal conductivity allowed the temperature of the support ring to adjust rapidly in response to changes in the yoke iron temperatures. The temperature change in the servo circuit was marginally faster compared to the average temperature change in the shell. This phenomenon primarily resulted from the relatively low heat conduction efficiency between the servo circuit and the air gap, leading to rapid internal energy accumulation. The heat conduction efficiency between the pendulum assembly and other components was relatively low, resulting in a significant temperature gradient between the pendulum assembly and the shell. The change in $\Delta T_{MS}$ was initially slightly greater than $\Delta T_{TOR}$ but became consistent with $\Delta T_{TOR}$ in the later stages. The temperature gradient between the entire meter head and the shell was marginally larger compared to that between the internal components (such as the magnetic steel and yoke iron) of the meter head and the shell. This discrepancy was primarily attributed to the inclusion of the internal pendulum assembly in the overall temperature calculation of the meter head, which exhibited a higher temperature gradient relative to the shell, thereby increasing the overall temperature gradient between the meter head and the shell.

The results of the unsteady heat transfer simulation experiment for the QFA indicate that variations in external environmental temperature cause a gradual propagation of internal temperature changes, originating from the QFA shell and progressing inward. Prior to achieving thermal equilibrium, the internal temperature distribution exhibits spatial and temporal heterogeneity. When temperature changes occur, the primary components exhibiting temperature gradient within the QFA are the yoke iron, pendulum assembly, servo circuit, and magnetic steel assembly. These components critically determine the gain magnitudes of each transfer link in the closed-loop system of the QFA and significantly influence the temperature coefficient of key parameters. Therefore, in traditional temperature error correction methods, assuming uniform temperature across all components fails to eliminate errors caused by temperature gradients during unsteady heat conduction. When developing a temperature error correction method, it is crucial to focus on and mitigate the impact of temperature gradients between components on measurement accuracy.

## 5. Validation of the Heat Transfer Simulation Model for the QFA

### 5.1. Temperature Testing Platform for the QFA

**Figure 11:**
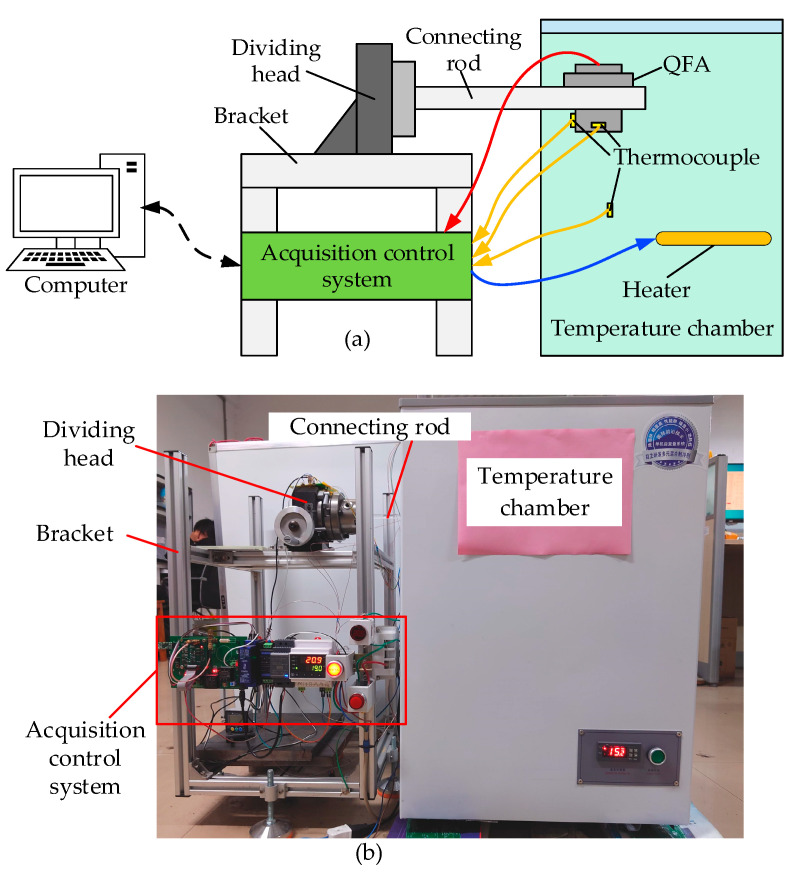
Temperature testing platform for QFA. (**a**) The structure of the temperature testing platform; (**b**) a physical map of the temperature testing platform.

To validate the accuracy of the heat transfer simulation model for the QFA, this study constructed a testing platform, as illustrated in Figure 11. The platform primarily consisted of a temperature chamber, an indexing head, a mounting bracket, a data acquisition controller, and a computer. The quartz connecting rod was linked to the external dividing head via the experimental operation port on the side of the temperature chamber, and the QFA was securely mounted at the front end of the connecting rod using a fixture. Thermocouples were affixed to the exterior of the QFA shell and the top surface of the gauge head to measure their respective temperatures. Among these, the thermocouple used for measuring the temperature of the meter head was inserted through a pre-drilled operation hole at the upper end of the QFA shell. This hole was originally reserved for welding the servo circuit and the lead wire of the meter head. The thermocouple was then bonded to the upper surface of the meter head. Additionally, thermocouples were positioned inside the temperature chamber to monitor the ambient temperature. The temperature control acquisition and control system was tasked with collecting output signals from the QFA as well as temperature data from the shell, meter head, and surrounding environment. Additionally, it controlled the heater within the temperature chamber to regulate the heating power. Data acquisition and control signals were transmitted wirelessly between the system and the computer, enabling the experimenters to remotely operate the experimental device. The testing platform operated within a temperature range of −45 °C to +80 °C, with a temperature adjustment accuracy of 0.2 °C, temperature uniformity of ±0.5 °C, and temperature measurement accuracy of 0.1 °C.

### 5.2. Analysis of Experimental Results on Temperature Variations in the QFA’s Shell and Meter Head

**Figure 12:**
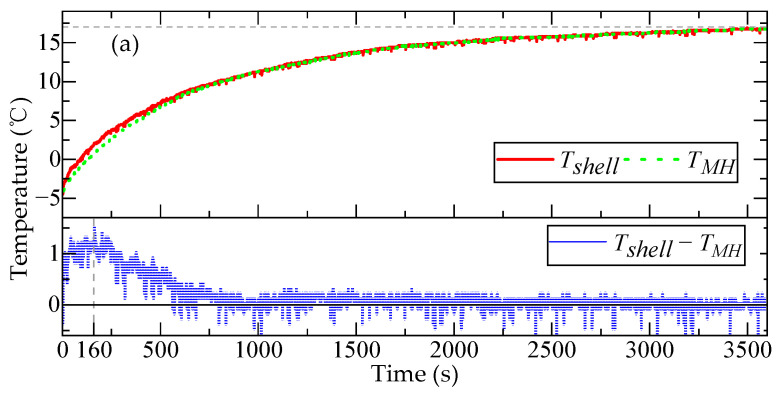

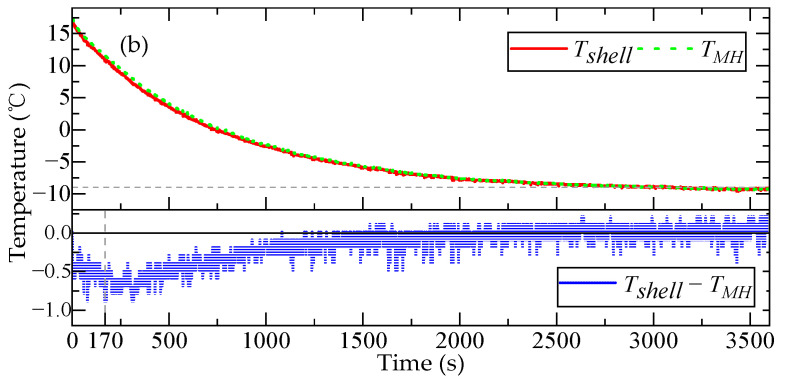
The variation curves of the temperature of the shell $T_{shell}$ and the upper surface temperature of the meter head $T_{MH}$ when the temperature changed. (**a**) Results observed when the ambient temperature rose from −4.3 °C to 17.3 °C; (**b**) results observed when the ambient temperature dropped from 17.3 °C to −9.7 °C.

To validate the accuracy of the heat transfer simulation model for the QFA, the fitting degree between the simulated temperatures of the shell and the meter head and the experimentally measured temperatures was analyzed and compared. Figure 12 illustrates the experimental results of the temperature change curves for the accelerometer shell temperature ($T_{shell}$) and the upper surface temperature of the meter head ($T_{MH}$) within 3600 s after the temperature change.

The overall variation trend of the temperature of the shell was consistent with that of the upper surface temperature of the meter head. Under a heating environment, from 0 to 1000 s, the temperature change in the meter head lagged behind that of the shell. Specifically, between 0 and 160 s, the temperature gradient between $T_{shell}$ and $T_{MH}$ gradually increased; from 160 to 1000 s, this temperature gradient gradually decreased, with the maximum difference reaching approximately 1.5 °C. After 1000 s, the temperature changes in $T_{shell}$ and $T_{MH}$ became synchronized. Under a cooling environment, within the initial 0 to 1500 s, the temperature change in the meter head lagged behind that of the shell. From 0 to 170 s, the temperature gradient between $T_{shell}$ and $T_{MH}$ gradually increased; from 170 to 1500 s, this temperature gradient gradually decreased, reaching a maximum difference of approximately 1.0 °C. After 1500 s, the temperature changes in $T_{shell}$ and $T_{MH}$ became synchronized. The measured variation curves of $T_{shell}$ and $T_{MH}$ aligned with the theoretical analysis and simulation experiment results regarding the internal temperature changes in the accelerometer. This indicates that the temperature response of the meter head lagged behind that of the shell.

**Figure 13:**
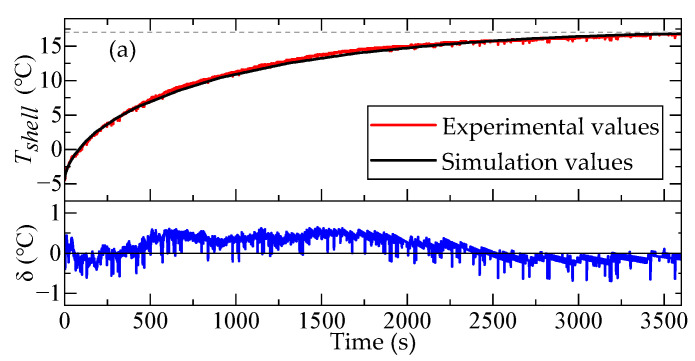

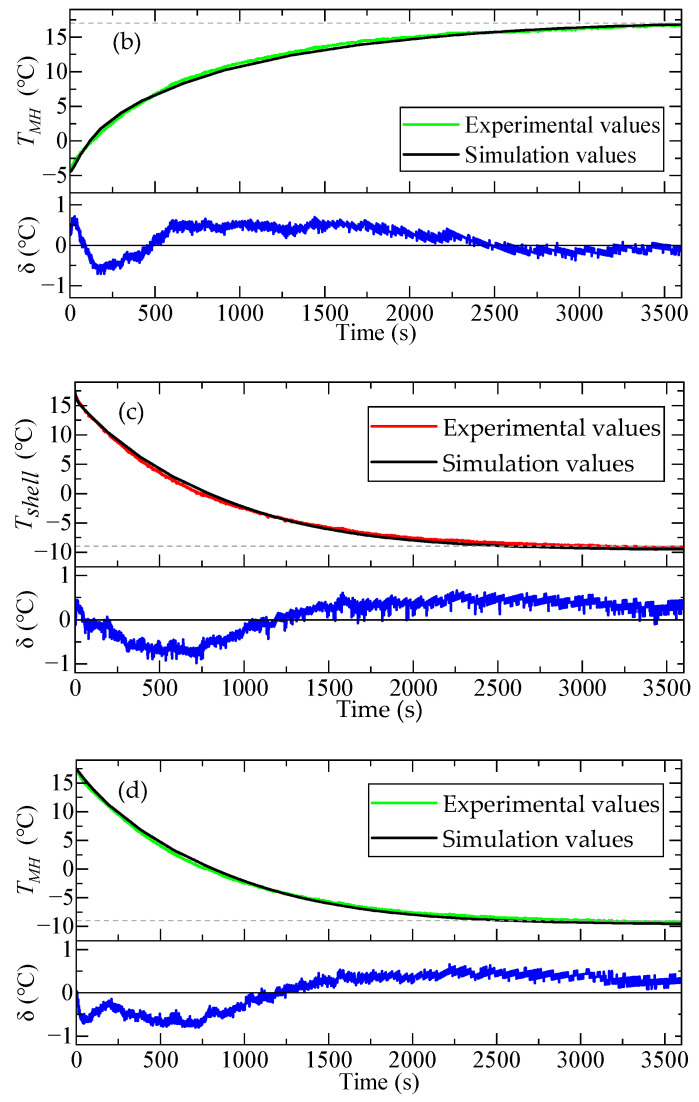
Comparison of simulation results and experimental results for temperature variation curves of $T_{shell}$ and $T_{MH}$ under ambient temperature changes. (**a**) Comparison of experimental and simulation values of $T_{shell}$ as ambient temperature increased from −4.3 °C to 17.3 °C; (**b**) comparison of experimental and simulation values of $T_{MH}$ as ambient temperature increased from −4.3 °C to 17.3 °C; (**c**) comparison of experimental and simulation values of $T_{shell}$ as ambient temperature dropped from 17.3 °C to −9.7 °C; (**d**) comparison of experimental and simulation values of $T_{MH}$ as ambient temperature dropped from 17.3 °C to −9.7 °C.

The simulation and experimental results of the temperature variation curves for the accelerometer shell temperature $T_{shell}$ and the upper surface temperature $T_{MH}$ of the meter head were compared as the ambient temperature varied, as illustrated in Figure 13. In this figure, δ represents the discrepancy between the simulated and measured values. As shown in Figure 10, the trends of the simulation values and experimental values were consistent. The discrepancy between the simulation and experimental results remained within ±1 °C. These simulation results accurately captured the temperature variations in the QFA.

Table 4 lists the three error evaluation metrics, namely, the Mean Absolute Error (MAE), Maximum Error (MAXE), and Root Mean Square Error (RMSE), comparing the simulation values with the experimental values of the temperature variation curves for $T_{shell}$ and $T_{MH}$. The results demonstrate that the simulation values exhibited a high degree of agreement with the experimental values, with an average RMSE of merely 0.4 °C. This indicates that the heat transfer simulation model for the accelerometer can accurately predict the distribution patterns of the internal temperature field, making it a reliable platform for simulating and analyzing the temperature characteristics of internal components, as well as establishing their temperature models.

## 6. Conclusions

This paper focused on the study of the unsteady heat conduction process in a QFA. Through simulation and experimental analysis, we investigated the temporal and spatial variation laws of the temperature field within these devices. The research findings indicate that the thermal properties of internal components significantly influence the transient thermal behavior. Specifically, when the external environmental temperature changes, a lag occurs in the internal temperature response of the QFA. Prior to achieving thermal equilibrium, there is both a time delay and spatial non-uniformity in the internal temperature distribution relative to the external temperature. The temperature gradient between the accelerometer shell and the internal meter head is predominantly observed during the initial phase of unsteady heat conduction, exhibiting an initial increase followed by a subsequent decrease. Significant temperature gradients are also present within the yoke iron, pendulum plate assembly, servo circuit, and magnetic steel assembly during this unsteady heat conduction phase. These components are the primary factors contributing to substantial deviations in the current temperature correction method during unsteady heat conduction. The experimental results demonstrate that the average RMSE of the QFA heat transfer simulation model established in this study is merely 0.4 °C, indicating a high degree of accuracy in simulating the actual temperature variations in the QFA. This model can serve as an effective experimental approach for developing temperature prediction models for internal components. Furthermore, it provides essential technical support for establishing temperature-based error correction methods for the internal components of the accelerometer, thereby reducing correction errors during non-steady-state heat conduction.

## Figures and Tables

**Table 1:** Principal geometric dimensions of the QFA (mm).

Geometric Name	Value (mm)	Geometric Name	Value (mm)
Shell height	20.80	Pendulum reed thickness	0.72
Shell diameter	25.40	Pendulum reed diameter	17.50
Meter head height	16.16	Coating area of pendulum reed	74.66 (mm^2^)
Meter head diameter	23.50	Length of flexible beam	2.10
Torquer coil height	2.40	Width of flexible beam	3.00
Torquer coil diameter	10.60	Thickness of flexible beam	0.03
Yoke iron gap	0.02	Flexible beam spacing	3.46

**Table 2:** Thermal physical property parameters of materials for each component of the QFA.

Part Name	Material Model	Density ρ (kg/m^3^)	Heat Capacity c [J/(kg·K)]	Thermal Conductivity λ [W/(m·K)]	Thermal Diffusivityα (m/s^2^)
Shell	1Cr18Ni9Ti	7900	502	16.0	4.03 × 10^−6^
Quartz pendulum	JGS1	2200	772	1.40	8.24 × 10^−7^
Pendulum coating	Au	19,300	134	296.4	1.15 × 10^−4^
Torquer coil	Cu	8900	385	386.4	1.13 × 10^−4^
Coil frame	Al_2_O_3_	3900	780	20.0	6.57 × 10^−6^
Magnetic steel	XGS240/46	7300	467	95.0	2.79 × 10^−5^
Magnetic pole piece	1J50	8020	455	81.1	2.22 × 10^−5^
Compensation ting	1J38	8020	522	20.0	4.78 × 10^−6^
Yoke iron	4J36	8100	515	11.0	2.64 × 10^−6^
Isolation ring	Al_2_O_3_	3900	780	20.0	6.57 × 10^−6^
Epoxy resin adhesive	DG-3S	1200	800	0.23	4.03 × 10^−6^
Fill gas	Air	1.29	1007	0.024	1.88 × 10^−5^

**Table 3:** Identifiers for each internal component of the QFA.

Component Name	Identifier	Component Name	Identifier
Torquer	TOR	Magnetic steel	MS
Servo circuit	SC	Yoke iron	YI
Spacer ring	SR	Meter head	MH
Pendulum reed	PR		

**Table 4:** Evaluation of simulation error for temperatures $T_{shell}$ and $T_{MH}$.

Evaluation Index	Heating Process		Cooling Process		Average Values
	$T_{shell}$	$T_{MH}$	$T_{shell}$	$T_{MH}$	
MAE (°C)	0.3558	0.3633	0.3591	0.3672	0.3613
MAXE (°C)	0.9946	0.8228	0.9809	0.8086	0.9017
RMSE (°C)	0.3957	0.3983	0.3969	0.3997	0.3976

## Data Availability

Data will be made available on request.
